# Plant Health and Sound Vibration: Analyzing Implications of the Microbiome in Grape Wine Leaves

**DOI:** 10.3390/pathogens10010063

**Published:** 2021-01-12

**Authors:** Birgit Wassermann, Lise Korsten, Gabriele Berg

**Affiliations:** 1 Institute of Environmental Biotechnology, Graz University of Technology, Petersgasse 12, 8010 Graz, Austria; birgit.wassermann@tugraz.at; 2 Department of Plant and Soil Sciences, University of Pretoria, Pretoria 0002, South Africa; lise.korsten@up.ac.za

**Keywords:** *Vitis vinifera*, grapevine, microbiota, phyllosphere, synthetic sound vibration, terroir, resilience

## Abstract

Understanding the plant microbiome is a key for plant health and controlling pathogens. Recent studies have shown that plants are responsive towards natural and synthetic sound vibration (SV) by perception and signal transduction, which resulted in resistance towards plant pathogens. However, whether or not native plant microbiomes respond to SV and the underlying mechanism thereof remains unknown. Within the present study we compared grapevine-associated microbiota that was perpetually exposed to classical music with a non-exposed control group from the same vineyard in Stellenbosch, South Africa. By analyzing the 16S rRNA gene and ITS fragment amplicon libraries we found differences between the core microbiome of SV-exposed leaves and the control group. For several of these different genera, e.g., *Bacillus, Kocuria* and *Sphingomonas*, a host-beneficial or pathogen-antagonistic effect has been well studied. Moreover, abundances of taxa identified as potential producers of volatile organic compounds that contribute to sensory characteristics of wines, e.g., *Methylobacterium*, *Sphingomonas*, *Bacillus* and *Sporobolomyces roseus*, were either increased or even unique within the core music-exposed phyllosphere population. Results show an as yet unexplored avenue for improved plant health and the terroir of wine, which are important for environmentally friendly horticulture and consumer appreciation. Although our findings explain one detail of the long-term positive experience to improve grapevine’s resilience by this unusual but innovative technique, more mechanistic studies are necessary to understand the whole interplay.

## 1. Introduction 

Plants and their associated microbes have been interacting with each other for a long time, forming assemblages of species that is referred to as a holobiont [1,2]. The composition of the plant microbiota varied during plants’ life cycle and is vertically transmitted [3]. Plant-associated microorganisms trigger important processes in plants, e.g., germination, circadian and annual cycles, fruit and seed formation and significantly contribute to plant health [4]. Interestingly, each healthy plant microbiome contains potential pathogenic microorganisms and their antagonistic counterparts, which together form a balanced functional network [5]. Antagonistic mechanisms, which convey this balance, are used for a long time to biologically control pathogens (reviewed [6,7]). The mode of action mediating antagonism includes (i) inhibition of microbial growth by diffusible antibiotics and volatile organic compounds (antibiosis), (ii) competition for colonization sites and nutrients, (iii) competition for minerals, (iv) degradation of pathogenicity factors of the pathogen such as toxins and (v) parasitism and lysis that involve production of extracellular cell wall-degrading enzymes such as chitinases and β-1,3-glucanase [8]. In addition, plant-associated microorganisms are able to directly activate the plant defense system by induced systemic resistance (ISR), which sometimes overlaps partly with that of pathogen-induced systemic acquired resistance (SAR); both ISR and SAR represent a state of enhanced basal persistence of the plant that depends on the signaling compounds jasmonic acid and salicylic acid [9].

Recent studies have shown that, in addition to plant-associated microorganisms, plants are also responsive towards natural and synthetic sound vibration (SV) by perception and signal transduction and activating ISR [10]. This resulted in resistance towards plant pathogens such as *Botrytis cinerea* [11], and improved plant health [12]. Moreover, novel data provide increasing evidence of a molecular mechanism for sound perception and transduction, improving germination, growth, development, crop yield and increased tolerance to drought stress [13,14,15]. Plants respond to the chewing sound of insect larvae, the buzz pollination of bees [16,17], and bat-dependent plants evolved acoustic reflectors for the bat echolocation system to attract their pollination partners [18]. These observations confirm acoustic communication beyond kingdoms with beneficial implications for the plant. Even unicellular organisms are suggested to respond in growth, metabolism, antibiotic and stress tolerance, when subjected to SVs [19,20,21], but to date, no study investigating the effect on the entire microbiota. Our hypothesis was that SV targets not only pathogens, it shifts the whole microbiome into a balanced state, which mediated better health and plant traits.

To verify this hypothesis, we used grape (*Vitis vinifera* L.) as a model. The indigenous microbiome [22,23,24,25,26] and pathogens [27] are well studied. The grape-associated microbiota was found to be involved in plant health [28]; and, by their volatile organic compounds on the vitivinicultural terroir [29,30,31,32,33,34]. Currently, conventional and organic grape production depend on high amounts of pesticides, which have to be drastically reduced [35]. Physico-stimulants like SVs, which have not been investigated on grapevine- or any other plant-associated microbiota, offers an environmentally friendly possibility to control pathogens. For the purpose of evaluating an effect of SVs in form of audible sound on native phyllosphere microbiota, we examined grapevine leaves (cultivar “Syrah”) from the vineyard De Morgenzon in Stellenbosch, South Africa (https://demorgenzon.com/music/) that was continually exposed to classical, mainly baroque music for the duration of the whole growing season, and compared them to non-exposed leaves. This pilot study proposes an effect of SV in form of music on the phyllosphere microbiome of grapevines, potentially supporting plant resilience and intensifying the terroir of red wine.

## 2. Results

### 2.1. The General Bacterial and Fungal Structure of the Grapevine Phyllosphere

Quality filtering using the DADA2 algorithm, removal of chimeric sequences and additional removal of mitochondrial and chloroplast sequences from the 16S rRNA gene fragments, yielded a 16S rRNA dataset containing 108,450 paired reads, assigned to 844 features and an internal transcribed spacer (ITS) region dataset consisting of 740,348 paired reads, assigned to 337 fungal features. Datasets were rarefied to 1205 bacterial and 30,298 fungal sequences, according to the sample with the lowest number of reads. Both the “Music” and the “Control” samples were dominated by Proteobacteria (61% and 45%, respectively), followed by Firmicutes (19% and 32%, respectively) and Actinobacteria (16% and 19%, respectively). Among dominating bacterial classes, “Music” samples were composed of Gammaproteobacteria (51%), Actinobacteria (14%), Bacilli (14%) and Alphaproteobacteria (9%). Similarly, “Control” samples contained the same classes with abundances of 36%, 17%, 26% and 8%, respectively. The fungal composition was found to be less consistent between the two groups. Here, “Control” samples were composed of 94% Ascomycota and 6% Basidiomycota, with the dominating classes Dothideomycetes (93%) and Tremellomycetes (5%). Exposure to music resulted in decreased abundance of Ascomycota (77%) and an increase in Basidiomycota abundance (22%); among them, Dothideomycetes and Tremellomycetes dominated again with 76% and 19%, respectively. 

**Figure 1:**
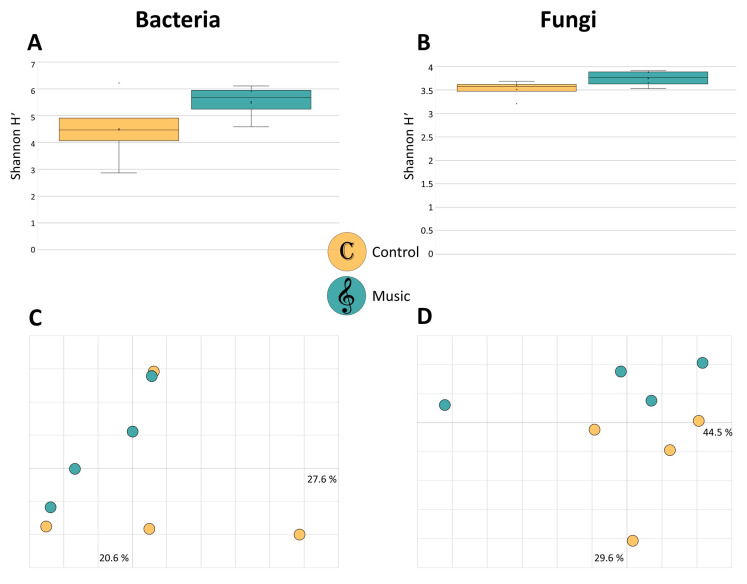
Alpha and beta diversity comparisons of grapevine leaf-associated bacterial and fungal communities. “Control” and “Music” samples are depicted in yellow and blue, respectively, as indicated by the legend in the figure center. Box-and-whiskers-plots represent bacterial (**A**) and fungal (**B**) alpha diversity, based on Shannon H′ index. Color-coded two dimensional Bray Curtis Principle Coordinates Analysis (PCoA) plots visualize community clustering of bacterial (**C**) and fungal (**D**) composition. Differences in alpha and beta diversity were not significant between the two groups, according to the Kruskal–Wallis test and analysis of similarity (ANOSIM) (*p* < 0.05), respectively.

In order to evaluate differences of microbial diversity within SV-exposed and non-exposed grapevine leaves, alpha and beta diversity analyses were observed for the whole dataset. We found no statistically significant difference in alpha diversity for the bacterial and fungal community; however, for both groups Shannon H′ index was higher in classical music-exposed leaves (Figure 1A,B). Beta diversity analyses, based on Bray Curtis dissimilarity matrix and assessed via pairwise analysis of similarity (ANOSIM), also revealed no significant differences for bacteria (Figure 1C) and fungi (Figure 1D). 

### 2.2. SV-Induced Differences in Phyllosphere Core Microbiomes Comprises Indicator Taxa for Resilience and Terroir

**Figure 2:**
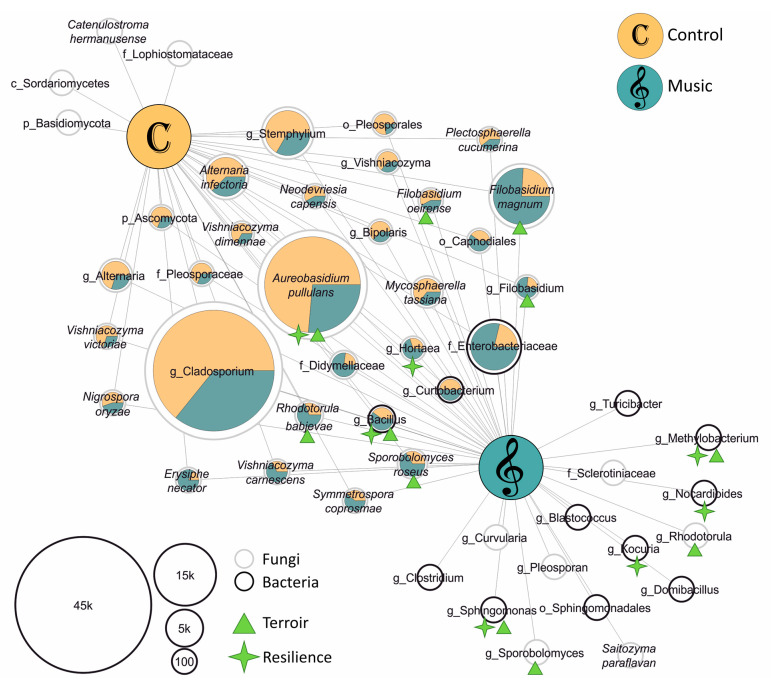
Shared and specific microbial taxa in sound vibration (SV)-exposed and control grapevine leaves. Only bacterial and fungal taxa (at species level) detected in at least 75% of the sample replicates were included in the network analysis. Node size corresponds to absolute abundance in the dataset and node outlines indicate bacteria (black) and fungi (grey) as indicated in the legend on the lower left. Pie charts of shared nodes indicate observed abundance within the two groups, where color of shared nodes is related to “Control” leaves (yellow) and “Music” leaves (blue). Green symbols attached to nodes point to the microbiota’s potential for supporting host resilience (asterisks) and/or contributing to the terroir (triangle).

In order to evaluate taxonomic composition changes induced by classical music, differential abundance based on the DESeq2 algorithm was tested on the entire feature table, resulting in no significant differences for taxa related to “Music” or “Control”. Thus, core microbiota were defined for “Music” and “Control” groups using features that were present in 75% of the replicates of the respective group. Core microbiota were assigned to 12 bacterial and 39 fungal taxa at species level, serving as a matrix for network analyses (Figure 2). In total, 26 fungal and 3 bacterial species were shared by “Music” and “Control” groups; their total abundance within the respective group is indicated via pie charts in the network. Fungal microbiota were present in both “Control” (four) and “Music” (six) core networks. In contrast, core network bacteria (nine) were unique to classical music-exposed leaves. Accordingly, fungal and bacterial communities exposed to classical music exhibited a higher degree of homogeneity compared to “Control” leaves. 

Next, we were interested in the potential impacts of the detected microbiota on plant health or the terroir. For that purpose, phyllosphere core taxa were subjected to literature research, revealing such functions for a total of 14 different taxa (indicated via green asterisks and triangles in Figure 2). Among them, only two (*Aureobasidium pullans* and *Filobasidium oeirense*) were more abundant in “Control” than in “Music” samples; however, both microorganisms were core members of the “Music” phyllosphere. The remaining 12 bacteria and fungi were either more abundant, or even unique within the core of SV-exposed leaves. Referring to current literature, grapevine-associated bacteria *Kocuria* and *Nocardioides* and the fungus *Hortaea* possess antagonistic properties towards necrotrophic *Botrytis cinerea* [36,37,38]. *Methylobacterium* and *Sphingomonas* strains counteract abundances of *Candidatus phytoplasma*, the causal agent of grapevine yellows [39], and several strains from the genus *Bacillus* are promising protective agents during heat and drought stress [22,40,41]. Regarding preferable impacts of microbiota on wine characteristics, abundances of *Methylobacterium, Sphingomonas* and *Bacillus* are positively correlated with typical sensory metabolites in finished wines [42]. *Sporobolomyces roseus* and *A. pullans* were identified to produce a broad spectrum of volatile organic compounds (VOCs) that are typical for wine terroir [29]; the same taxa, and the yeast genera *Sporobolomyces*, *Rhodotorula* and *Filobasidium,* are important participants of alcohol fermentation and the formation of the typical aromatic properties of wine [43,44,45].

## 3. Discussion

The present pilot study is the first describing potential impact of SVs in the form of music on the native plant microbiota. Our results suggest that perpetual exposure to music modulates the grapevine phyllosphere microbiota, demonstrating either increased abundance of specific bacteria and fungi, and under certain conditions, distinct taxa previously characterized for exhibiting beneficial characteristics in host resilience and/or wine terroir. This verified our hypothesis that SV induce a microbiome shift into a balanced state, which mediated better health and plant traits.

Among the latter, “Music”-associated taxa can be subdivided into the following groups: (i) their abundance correlates positively with the chemical composition and metabolite abundance of finished wines [42]; (ii) they have been identified to produce VOCs that are typical flavor compounds of red wine [29], and (iii) predominantly yeasts, that play important roles during alcohol fermentation, contributing significantly to quality and aromatic profiles of the product [43]. It has to be mentioned that (ii) and (iii) are not mutually exclusive. In fact, the production of wine is a complex process that includes the impact of bacterial and fungal consortia on wine chemistry [42]. 

Here, we must also point out the limitations of this pilot study. Despite our effort, an entirely randomized sampling was not practical due to the given circumstances in the vineyard; in particular, the positions of the speakers. Thus, an effect of soil-related variability between sampling points cannot be excluded. Integrating a starting situation without music and a subsequent examination of manipulating factors would furthermore allow a more comprehensive analysis. In addition, our observations must be confirmed for other *Vitis* cultivars and vineyards and in order to draw any conclusion on the impact of SVs on the taste of finished wines, the berry microbiota must be analyzed as well.

Nevertheless, t studies of sound waves on plants [10,11], plant pathogens [10,12] and the total grapevine-associated phyllosphere microbiome uncovered in this study, showed that music potentially boosts plant health and resilience by activating a plant’s immune system, prompting a corresponding microbiome shift. Furthermore, just as reinoculated grapevine-endophytic fungi have the potential to be applied as a fine-tuning regulator for wine grapes [46], we suggest music to be considered as a non-invasive agricultural management parameter that can augment the grapevine microbiome towards a favorable composition, thereby impacting the quality and characteristics of wine. However, the development of any microbiological application utilizing sound stimulation requires sufficient and in-depth fundamental research on the holobiont’s response to music. Detailed metabolomic analyses of the effect of specific SVs (hertz) and loudness (decibel) on the plant’s immune system are required to understand the underlying mechanisms. Nevertheless, this study suggests that SV in the form of music might impact the grapevine microbiome with yet unexplored potential for plant health and the terroir that is important for sustainable and environmentally friendly horticulture. 

## 4. Materials and Methods 

### 4.1. Experimental Design and Sample Processing

For the microbiome analyses, leaves of *Vitis vinifera* L. (cultivar “Syrah”) were collected in February 2019 from the vineyard De Morgenzon in Stellenbosch, South Africa. A subarea of the vineyard was planted in 2004 and equipped with loudspeakers, continually playing classical music (Baroque, and early Classic of selected composers) for 24 h daily and seven days a week over the whole growing season. More information about the background you can find here: https://demorgenzon.com/music/. According to the vineyard’s owners, after seven years of experience, “phenolic ripeness with lower sugar levels which results in wines with all the ripeness, fruit, and acidity one would want, but with slightly lower alcohol levels” were observed. For SV-exposed grapevine leaf samples (henceforth referred to as “Music”) four different *Vitis* plants, growing in close proximity to four different loudspeakers were selected. Each of the four replicates consisted of five randomly selected leaves, carefully preventing an effect of light exposure. Similarly, negative control samples (“Control”) were taken from the same orchard, and at the same day and time, but most distantly located to the speakers, where no music was audible. Aerial photograph from Google maps [47] indicating sampling points for “Music” and “Control” replicates are provided in the Supplementary Materials (Figure S1). No visible difference between leaves of “Music” and “Control” plot was observed during the sampling. Leaf samples were placed on ice immediately until processing under sterile conditions in the laboratory. For each replicate a total leaf area of 780 cm^2^ was analyzed by placing leaves on a cardboard stencil covered with sterile freezer bags and the microbial community was acquired from leaves by a series of washing and sonication steps according to the method described by Ortega et al. [48]. The total microbial suspension of each replicate was centrifuged at 6170× *g* for 20 min. The supernatant was removed and the moist pellet was transferred to a 2 mL Eppendorf tube and centrifuged again at 16,000× *g* for 20 min. Pellets were stored at −70 °C until further DNA extraction.

### 4.2. Library Generation and Illumina MiSeq Sequencing of 16S rRNA Gene and ITS Regions

Total community DNA was extracted using the FastDNA Spin Kit for Soil (MP Biomedicals, Solon, OH, USA) according to the manufacturer’s instructions using a FastPrep Instrument (MP Biomedicals, Illkirch, France) for 30 s at 5 ms^−1^. The following primer combinations were used for Illumina amplicon sequencing: 515f-806r [49] and ITS1f-ITS2r [50] to amplify the 16S rRNA gene fragments and the ITS region, respectively, with three technical replicates per sample. Amplification of host plastid and mitochondrial 16S DNA was blocked by adding peptide nucleic acid (PNA) [51] clamps to the PCR mix. PCR for 16S rRNA gene amplification was performed in a total volume of 30  μL containing 5× Taq&Go (MP Biomedicals, Illkirch, France), 1.5  μM PNA mix, 0.25  mM of each primer, PCR-grade water and 1 μL template DNA. 16S rRNA gene fragment PCR was conducted using the following cycling conditions: 95  °C for 5  min, 30 cycles of 96  °C for 1  min, 78  °C for 5  s, 54  °C for 1  min, 74  °C for 60  s and a final elongation at 74  °C for 10 min. PCR mix for ITS region amplification contained 5× Taq&Go, 25  mM MgCl_2_, 10  μM of each primer, PCR-grade water and 1 μL template DNA, in a total of 20 μL. ITS region amplification was performed under the following cycling conditions: 95  °C for 5  min, 30  cycles of 94  °C for 30  s, 58  °C for 35  s, 72  °C for 40  s and final elongation at 72 °C for 10 min. A nested PCR step was carried out to add barcoded primers. The three technical replicates were combined and purified using Wizard SV Gel and PCR Clean-Up System (Promega, Madison, WI, USA). Samples were combined in equimolar concentration according to Nanodrop 2000 (Thermo Scientific, Wilmington, DE, USA) measurements, and then sequenced using Illumina MiSeq v2 (250  bp paired-end) amplicon sequencing.

### 4.3. Illumina MiSeq Data Processing and Statistics

Forward and reverse reads were joined in QIIME 1.9.1 and imported into QIIME 2 2019.1 where all downstream analyses were performed. Using the DADA2 algorithm, sequences were quality filtered, chimeric sequences were discarded and a feature table was constructed, containing sequence variants and representative sequences. Features were classified using a Naïve-Bayes feature classifier trained on Silva132 (16S rRNA gene) [52] and UNITE v7.2 (ITS) [53] databases. Mitochondria and chloroplast reads were discarded from 16S dataset. Core diversity script of QIIME 2 was applied to investigate alpha and beta diversity by rarefying feature tables to the lowest value of reads present in one sample. In order to evaluate taxonomic differences, core microbiomes were constructed, containing features present in 75% of the sample replicates. The OTU network was constructed on core bacterial and fungal species using Cytoscape version 3.5. [54]. Statistical analyses were performed in QIIME 1.9.1 and QIIME2 2019.1 using the Kruskal–Wallis test and analysis of similarity (ANOSIM) to test for significant differences in alpha (Shannon diversity) and beta (Bray Curtis dissimilarities) diversity, respectively.

## Data Availability

The raw sequence files supporting the findings of this article are available from the European Nucleotide Archive (ENA) at study Accession Number PRJEB37982.

## Supplementary Materials

The following are available online at https://www.mdpi.com/2076-0817/10/1/63/s1, Figure S1: Google maps images of sampling location.
