# Supplementary material for: Plant Health and Sound Vibration: Analyzing Implications of the Microbiome in Grape Wine Leaves
Source: Pathogens. 2021 Jan 12;10(1):63. doi: 10.3390/pathogens10010063 (PMC7828301; doi:10.3390/pathogens10010063)
Supplement: Supplementary file 1 [file pathogens-10-00063-s001.pdf]

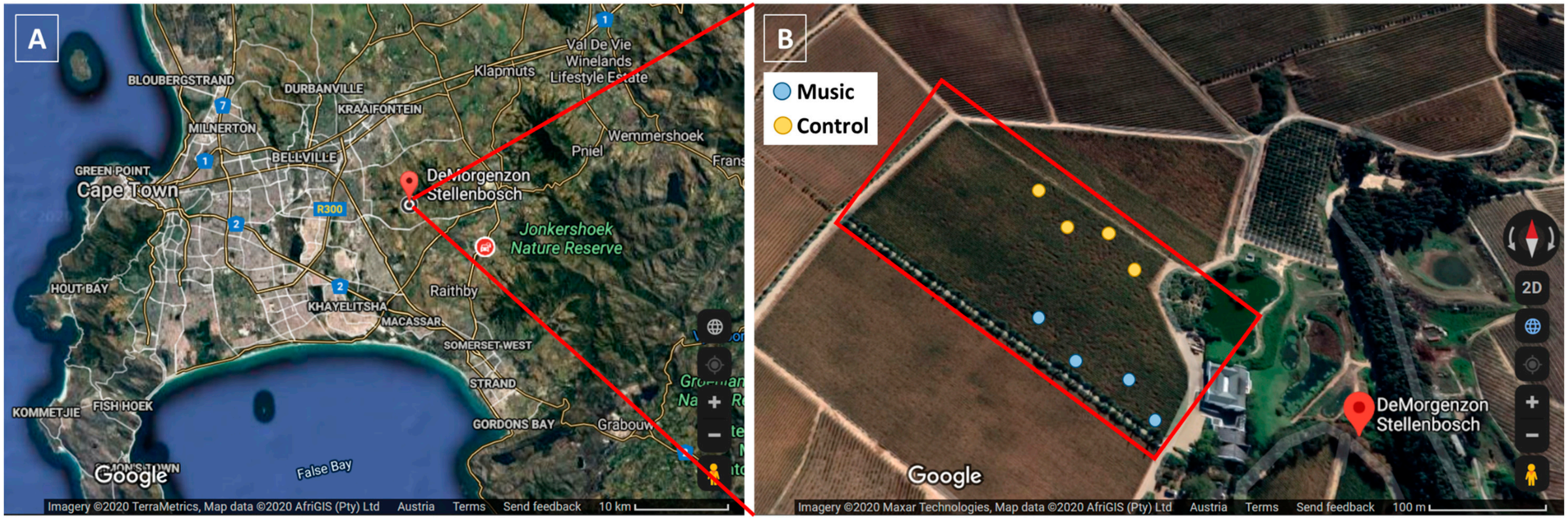

Figure S1. Google maps images of sampling location. (**A**) Location of the wine estate DeMorgenzon in Stellenbosch/South Africa (scale 1:416,666,667). (**B**) Detailed 3D map (scale 1:3448) of the location selected for collecting grapevine leaf samples, where the area of planted "Syrah" cultivar is indicated by a red square (40,000 m²). Sampling points of "Music" and "Control" replicates are indicated by blue and yellow dots, respectively, as described in the legend on the upper left of (**B**).
